# A critical analysis of eating disorders and the gut microbiome

**DOI:** 10.1186/s40337-022-00681-z

**Published:** 2022-11-03

**Authors:** Sydney M. Terry, Jacqueline A. Barnett, Deanna L. Gibson

**Affiliations:** 1 Department of Medicine, Faculty of Medicine, University of British Columbia, Okanagan Campus, Kelowna, BC Canada; 2 Department of Biology, I.K. Barber Faculty of Science, University of British Columbia, Okanagan Campus, Kelowna, BC V1V 1V7 Canada

**Keywords:** Feeding and eating disorders, Anorexia nervosa, Bulimia nervosa, Feeding behavior, Gastrointestinal microbiome, Mental health, Humans, Physiology, Dysfunctional immunity

## Abstract

**Abstract:**

The gut microbiota, also known as our “second brain” is an exciting frontier of research across a multitude of health domains. Gut microbes have been implicated in feeding behaviour and obesity, as well as mental health disorders including anxiety and depression, however their role in the development and maintenance of eating disorders (EDs) has only recently been considered. EDs are complex mental health conditions, shaped by a complicated interplay of factors. Perhaps due to an incomplete understanding of the etiology of EDs, treatment remains inadequate with affected individuals likely to face many relapses. The gut microbiota may be a missing piece in understanding the etiology of eating disorders, however more robust scientific inquiry is needed in the field before concrete conclusions can be made. In this spotlight paper, we critically evaluate what is known about the bi-directional relationship between gut microbes and biological processes that are implicated in the development and maintenance of EDs, including physiological functioning, hormones, neurotransmitters, the central nervous system, and the immune system. We outline limitations of current research, propose concrete steps to move the field forward and, hypothesize potential clinical implications of this research.

**Plain English summary:**

Our gut is inhabited by millions of bacteria which have more recently been referred to as “our second brain”. In fact, these microbes are thought to play a role in ED behaviour, associated anxiety and depression, and even affect our weight. Recent research has dove into this field with promising findings that have the potential to be applied clinically to improve ED recovery. The present paper discusses what is known about the gut microbiome in relation to EDs and the promising implications that leveraging this knowledge, through fecal microbiome transplants, probiotics, and microbiome-directed supplemental foods, could have on ED treatment.

## Overview

The gut microbiome has captured the attention of the medical field and has been implicated in a myriad of conditions including neuropsychiatric disorders, encompassing eating disorders (EDs) [1], metabolic disorders, and immune-mediated diseases. Research regarding EDs and the gut microbiome remains nascent and speculative, yet promising [2–9]. Here we provide a critical analysis of the field, suggest practical steps that can be taken to move the field forward, and discuss the potential implications of this research.

EDs are mental health disorders comorbid with physical and psychosocial disease; only about 50% of affected individuals achieve lifelong remission [10]. The DSM-5 outlines eight ‘feeding and eating disorders’ [11], however ED research disproportionally investigates anorexia nervosa (AN) and to a lesser degree bulimia nervosa (BN) and binge eating disorder (BED). The present paper will focus on AN, which can be subdivided into restrictive type (ANR) and binge-eating/purging type (ANBP), as well as BN. BED will not be discussed, because, while not the same, BED is highly correlated with obesity and a large body of literature already exists that explores the relationship between the gut microbiome and obesity [12]. Additionally, avoidant/restrictive food intake disorder (ARFID), an ED *not* driven by a desire to be thin, but instead by food avoidance/restriction due to sensory sensitivity, lack of interest, and fear of adverse consequences, will not be discussed due to a lack of current research. However it is important to note that ARFID is a disorder of gut-brain interaction and is likely influenced by some of the same gut microbiota-ED behaviour correlations as AN [13]. Furthermore, there is currently no approved mediations for AN or AFRID [14, 15], further supporting the need for research into the gut microbiome and AN and ARFID as this could lead to novel treatments.

The etiology of EDs is complex but includes genetic underpinnings, and indeed AN and BN display a genetic diathesis [16]. Recently, a genome wide association study identified eight significant loci for AN [17] and epigenetics has also been implicated in ED etiology [18]. Other biological, social, cultural, and psychological factors contribute to ED etiology [19], and gut microbes modulate a host of biological processes that affect the clinical manifestations of EDs—the details of which will be discussed in subsequent sections of this paper.

The gut microbiome refers to the 300–500 bacterial species inhabiting the human gastrointestinal system [20], and the dominate bacterial species are divided into three phyla: *Bacteroidetes, Firmicutes,* and *Actinobacteria* [21]. When studied in humans, obese individuals have more *Firmicutes* and almost 90% less *Bacteroidetes* than lean counterparts, and weight loss in the obese group is associated with a decrease in *Firmicutes* and increase in *Bacteroidetes* [22].

The gut microbiome changes over the course of the lifespan, as it is shaped by a multitude of factors including host genetics, age, and sex. Indeed, it is thought that development of the gut microbiome parallels that of brain development [23]. The gut microbiome is also influenced by diet, and in turn, the microbes regulate energy utilization, thus having implications on body composition. Obesity studies have revealed the gut microbiome is responsible for energy metabolism using twin fecal transplants in germ-free mice, revealing a causal role for microbes and energy harvest [24]. Indeed, macronutrient bioavailability is influenced by gut microbial metabolic processes [25]. Interestingly, short-chain fatty acids (SCFAs), produced from carbohydrate fermentation, may modulate glucose metabolism and fat deposition, and SCFAs are observed to be less abundant in AN populations compared to controls [9]. This may also reflect metabolic dysfunction observed in the microbiome of patients with AN, as perturbations in carbohydrate degradation and amino acid biosynthesis are observed [25].

**Fig. 1:**
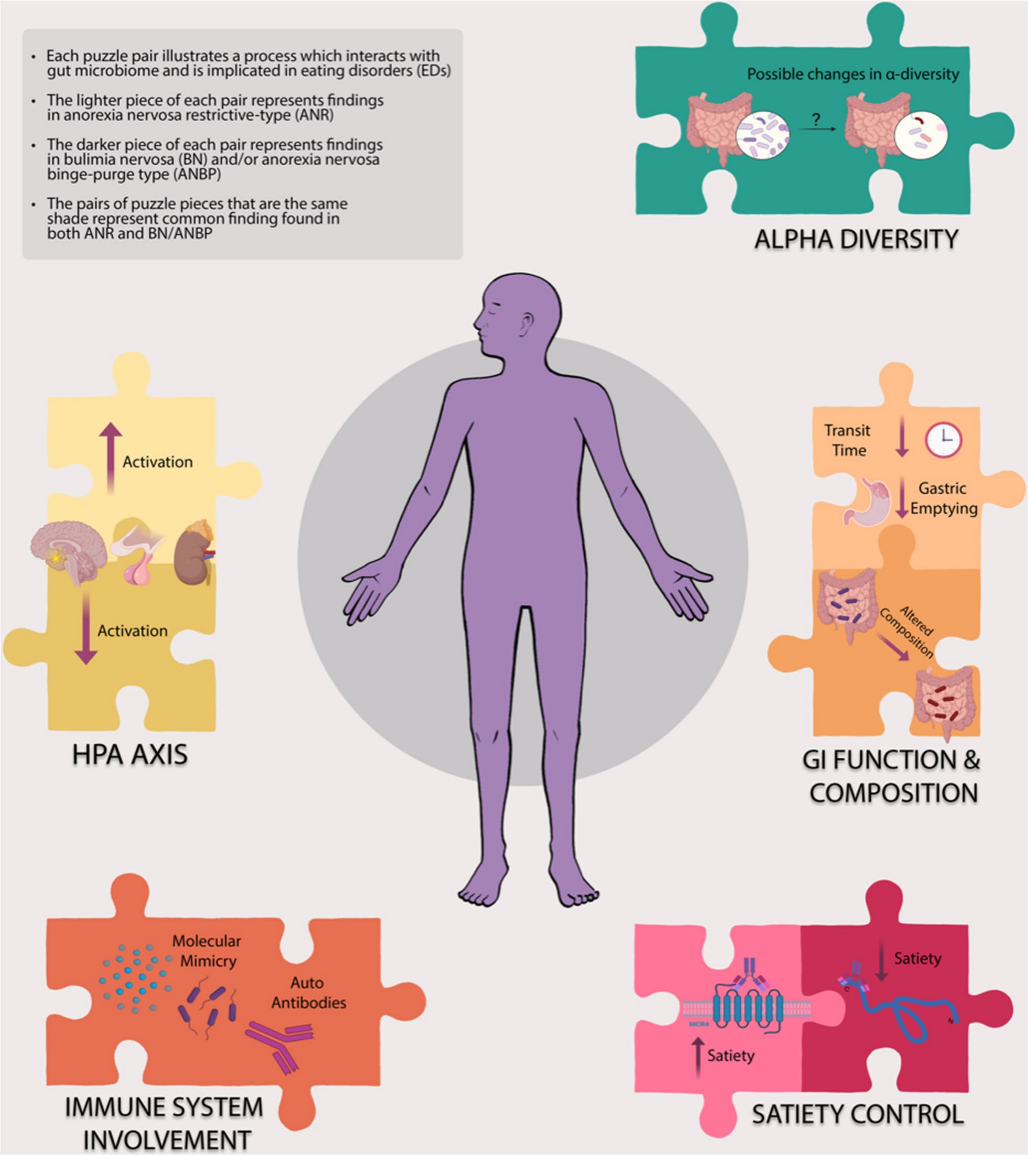
This figure summarizes the complex interplay between biological processes and gut microbes that are thought to be implicated in EDs, depicting the deeply interconnected nature of these relationships. Abbreviations: *GI—*gastrointestinal, *HPA*—hypothalamic–pituitary–adrenal

The diversity of microbes in a single ecological community is known as α-diversity, and is commonly assessed in research, with increased α-diversity correlated with better health [26]. Perturbations within the microbiome, or ‘gut dysbiosis’, are associated with disease, often resulting from an overgrowth of potentially harmful organisms, loss of beneficial organisms, and reduction in species diversity resulting in the loss of the normally tolerogenic and symbiotic relationship [27]. In particular, a decrease in diversity of gut microbiota, especially in bacterial species producing butyrate, appear to correlate with increased anxiety, depression and ED psychopathology [9]. The gut microbiota may be a missing piece of the ED puzzle as two main pillars, eating behaviour and mental health, are influenced by gut dysbiosis (Fig. 1).

## What does current research tell us?

### Individuals with EDs may have a distinct gut microbiome

The ED field is turning its attention towards the gut microbiota. Commonly, reduced α-diversity is seen in ED rodent populations compared to controls [3], however this finding is not consistent across studies. Some clinical research postulates that α-diversity is negatively correlated with ED psychopathology, including depression and weight/shape concerns [5]. Interestingly, this study found that individuals with AN demonstrated reduced α-diversity before and after hospital-based weight restoration when compared to healthy controls, however as the AN group gained weight with treatment, the bacterial composition of their gut microbiome became more similar to that of the control group. Although conclusions cannot be made based on one study, this area warrants further research.

Microbial α-diversity in relation to BN has yet to be explored, but the gut microbiome and metabolomics profile in ANR and ANBP has been investigated. Although no significant differences in α-diversity between ANR and ANBP are observed, women with ANBP demonstrate a higher abundance of *Bifidobacterium* spp. and *Odoribacter* spp., and relative decreases of *Haemophilus* spp., compared to women with ANR [6]. ANR, ANBP, and control groups display differences in fecal metabolites, with similarities found between ED groups, perhaps suggesting distinct gut microbial functions are associated with EDs [6]. ED groups have altered metabolites reflective of reduced energy metabolism including deoxycytidine, isoleucine, malic acid, n-acetyl-glucosamine, palmitic acid, rhamnose, sorbose, tagatose, and xylose while some specific metabolites, including rhamnose, xylose, deoxyadenosine, thionic acid, arabinose, acetic acid, lactose, gamma-aminobutyric acid, pyroglutamic acid, succinic acid, and scyllo-inositol are altered between the ANBP And ANR groups [6]. These findings may be reflective of nutritional aberrations resulting from ED behaviours relating to ANBP and ANR, and suggest that ED behaviours including binging/purging and restricting are related to distinct gut microbiome compositions. Rigorous research regarding α-diversity, gut microbiome composition, and metabolomic variation in EDs may provide more insight into the validity of these preliminary findings and subsequently may have the potential to inform ED etiology and symptomology.

### Gastrointestinal functioning affects the gut microbiome

Clinical manifestations of EDs are related to gastrointestinal (GI) functioning known to be influenced by gut microbe composition. Severe food restriction leads to delayed gastric emptying and a slower transit time, resulting in earlier satiety and bloating, reinforcing restrictive behaviour via physiological and psychological pathways [28]. These processes curate a specific GI environment, contributing to a distinct microbial profile. Additionally, slower transit time contributes to constipation, which in turn appears to be correlated with increased abundance of short-chain fatty acids in the gut microbiome [29]. Altered GI functioning affects gut microbial gene expression by disrupting circadian rhythms that govern their function [30]. Some gut microbes require the by-products of others to flourish, for example, butyrate producers need lactate produced by *B. adolescentis* [31], thus the effects of GI function on one gut microbe may have a cascading effect, on the entire community. *Escherichia coli* produces lipopolysaccharide that delays gastric emptying [32]. Research demonstrates that the intestinal microbiota of individuals with AN are enriched with Enterobacteriaceae, of which *Escherichia coli* is a member [7], and while this does not confirm or deny a relationship between altered GI function, AN and gut microbes, this observation is a springboard into further research. Additionally, the selection of gut microbes in individuals with AN and low adiposity may be an adaption that perpetuates AN pathology by providing the host with energy in a caloric-deprived environment, perhaps contributing to the high relapse rates observed in AN [33]. Furthermore, the nutrient-poor state associated with AN may lead to physiological changes including decreased small intestine surface area, and alterations to villus architecture, which reduce the gut’s absorptive capacity. This may pose difficulties with weight restoration and threaten recovery [34].

Purging behaviours also affect GI physiology and functioning, potentially resulting in damage to the mucosal lining, motility disturbances, and changes to gastric capacity and gastric emptying [35]. Many individuals with ANBP and BN purge through laxative misuse, which, depending on the frequency and quantity, may result in chronic diarrhea, electrolyte imbalances, and colonic motility impairment. Mice given laxative treatment show a 75% difference in gut bacterial taxa composition two weeks after cessation of treatment, a change mediated by host-dependent factors (colonic mucus loss and immune function) and host-independent factors (growth inhibition due to altered gastrointestinal osmolality) [36]. Over-exercise and self-induced vomiting are other purging behaviours not yet explored in relation to the gut microbiome. However, preliminary research into the relationship between exercise and the gut microbiota in the general population suggests that regular exercise is related to greater α-diversity, the gut microbiome composition changes in response to exercise regime,—but these changes are not sustained after 6 weeks of stopping the exercise regime, and the microbiota of lean individuals appears to be more influenced by an exercise intervention compared to the gut microbiota of overweight individuals [37].

### The hypothalamic–pituitary–adrenal axis and gut microbiome may be intimately intertwined

The hypothalamic–pituitary–adrenal (HPA) axis regulates metabolism, emotion, and stress and is implicated in EDs. In early life, gut microbes help shape the HPA axis, a process mediated by stress. Exposure to trauma and adverse events during critical periods of prenatal and early postnatal life interferes with colonization of the gut, increasing propensity towards mental health disorders, and dysregulated GI, metabolic, and immune processes [38]. In rodents, early life stress induced by maternal separation results in dysbiosis with specific reductions in *Lactobacillus* spp. [39]. Chronic stress later in life affects the gut microbiome forming an axis with the HPA system leading to anxiety-like behaviours [40]. HPA axis dysregulation is implicated in both AN and BN [41]. In fact, AN is considered a state of functional hypercortisolism, resulting from hypersecretion of corticotrophin-releasing hormone (CRH), the primary regulatory hormone of the HPA axis. CRH is a powerful anorexic agent that likely mediates starvation behaviour in AN. Conversely, BN is associated with reduced plasma cortisol, and consequently reduced satiety, likely exacerbating bingeing behaviour [41]. Thus, early life stress may be a predisposing factor for ED, through its role in shaping the HPA-axis and subsequent consequences on hunger and satiety cues.

### The gut microbiome interacts with neurotransmitter activity

The melanocortin system (MC) system is composed of MC peptides, MC receptors, endogenous antagonists, and ancillary proteins which together play a role in energy homeostasis, inflammation, pigmentation, and sexual function [42]. In the case of EDs, increased MC system activity causes dysregulated neurotransmitter signalling, notably of serotonin and dopamine. Serotonin is synthesized from tryptophan, an essential amino acid obtained from food, in both the brain and the gut. Under physiological conditions, serotonin has many roles as its receptors are found throughout the body. Notably, serotonin regulates smooth muscle in the gastrointestinal systems and aids in digestion, as well it has been implicated in mood regulation and has been colloquially termed the “feel good” chemical [43]. Altered neurotransmitter activity affects feeding and behavioural aspects of EDs. Increased binding of the serotonin receptor 1A (5-HT_1A_) occurs in individuals with EDs affecting satiety, impulse control, and moods [44]. Serotonin promotes food restriction, a behaviour which reduces anxiety in individuals with AN, and thus increased binding of the 5-HT_1A_ receptor promotes negative post-prandial affect in individuals with AN [45]. Additionally, decreased serotonin signaling contributes to bingeing observed in BN [8]. A blunted dopamine response is associated with reduced food intake in AN [46], but with bingeing in BN [47].

Gut microbes modulate the host’s neurotransmitter activity and produce neurotransmitters autonomously [48], yet this has not been explored in relation to EDs. Several neurotransmitters like serotonin, indoles, and kynurenines are regulated by tryptophan metabolism which is influenced by the gut microbiome. Indeed, inadequate nutrition has been correlated with a decreased concentration of kynurenic acid in the cerebral spinal fluid of individuals with AN, however the clinical consequence of this remain unclear [49]. Additionally, *Bifidobacterium* spp*.* are instrumental in maintaining homeostasis between kynurenine and tryptophan production [50] and individuals with AN have reduced *Bifidobacterium* spp. [51]. While no conclusions can be made yet, the relationship between gut microbes and neurotransmitters in EDs warrants future investigation.

### The gut microbiome interacts with hunger hormones

Hunger and satiety hormones including, leptin, ghrelin, peptide YY (PYY) and neuropeptide Y (NPY) are implicated in ED behaviours may be affected by gut microbes. Under normal physiological conditions, leptin inhibits hunger via a negative feedback mechanism, and ghrelin works in opposition to stimulate hunger [52]. Like leptin, PYY has anorexigenic, proprieties and it is secreted in proportion to caloric intake, and like ghrelin, NPY stimulates food intake [52]. When studied in rodents, leptin is positively correlated with the quantity of *Bifidobacterium* spp. and *Lactobacillus* spp., and negatively correlated with the quantity of *Clostridium* spp., *Bacteroides* spp., and *Prevotella* spp. Conversely, ghrelin levels are negatively correlated with abundances of *Lactobacillus* spp., and positively correlated with abundances of *Bacteroides* spp. [53]. Significant weight loss, characteristic of AN, leads to lower leptin levels and higher ghrelin levels. These observations could provide clues into the potential role gut microbes may hold in ED behaviours. Further adding to this, the immune system is likely implicated in the relationship between gut microbes and hormones. Human serum contains IgG and IgA autoantibodies against appetite-regulating peptides, including leptin, ghrelin, PYY, and NPY [54]. These autoantibodies cross the blood–brain barrier and interact with hunger centres, including the arcuate nucleus. Sequence homology is observed between peptide hormones and gut microbes including *Lactobacillus* spp, *Bacteriodes* spp, *Helicobacter pylori*, *E. coli*, and *Candida* spp., suggesting the gut microbes, through molecular mimicry, may impact feeding behaviour.

### The gut microbiome may affect hunger and satiety through interactions with the immune system

Connections between the immune system, central nervous system (CNS), and gut microbes may explain satiety differences observed in AN and BN. The CNS contributes to abnormal feeding behaviour, in part, through the MC system. The MC type 4 receptor (MC4R) is implicated in feeding, mood, and emotional regulation, and the MC system shows increased activity in individuals with EDs [2]. Stimulation of the MC4R induces anorexia while blocking it leads to hyperphagia. Additionally, stimulation of the MC4R is correlated with higher levels of anxiety, a trait commonly comorbid with EDs [2].

Gut microbes influence MC activity via an immune-mediated pathway. *E. coli* produces caseinolytic protease B (ClpB), a heat-shock disaggregation chaperone protein which is a molecular mimic of α-MSH, the primary MC4R activating ligand. ClpB forms immune complexes (IC) with α-MSH-reactive IgG (α-MSH/IgG IC), which bind the MC4R and activate the MC system [55]. Indeed, plasma concentrations ClpB are significantly increased across ED groups compared to controls, and these increased levels are correlated with increased EDI-2 scores [56]. A lower BMI is correlated with a higher prevalence of *E. coli* in the gut [57] and AN and BN populations display increased plasma α-MSH-reactive IgG levels compared to controls [58]. IgG generally binds the central portion of α-MSH, however variation in binding location is seen between AN and BN populations, and is implicated in MC4R signaling variation [2]. The C-terminal of α-MSH is essential for α-MSH to bind to MC4R, thus if IgG binds the C-terminal in the α-MSH/IgG IC, MC4R cannot be activated, and satiety would not be induced. This pattern of binding is seen in BN, but never in AN, and could explain a reduced satiety response in BN, but enhanced response in AN. An epitope shift of the α-MSH/IgG IC may contribute to an individual switching from AN to BN behaviours over the course of their ED [9]. Moreover, α-MSH/IgG IC binds and activates the MC4R at a lower threshold than α-MSH alone, further impacting the starvation behaviour [58]. When studied in rodents, stress is associated with an increase in ClpB production, thus physiological stress resulting from starvation may amplify this process [59].

### Experimental treatments leveraging the gut microbiome

While no approved treatments that leverage the gut microbiome exist for EDs yet, experimental treatments involving fecal microbiota transplantations (FMT), tailored probiotic supplements, and microbiome-directed supplemental foods are being investigated. Two case studies explore FMT for ED treatment. In one case, a 26-year-old female, who after clinical recovery from AN failed to maintain a healthy bodyweight (her BMI settled at 15 despite a 2500 kcal diet), received a FMT which resulted in weight gain of 13.6% over 36 weeks, with no negative side effects reported [60]. Additionally, 37-year-old female with a 25-year history of severe and enduring AN and more recent co-occurring small-intestinal bacterial overgrowth (SIBO) received a FMT from a healthy 67-year-old, female, first degree relative. The patient maintained a BMI of 17.4–18.4 over the 12-months following the FMT, and 1-year post-FMT she reports digestion complaints and restricts to almost no intake [61]. While these cases illustrate the potential therapeutic role of leveraging the gut microbiome, they also illustrate the complexity of ED treatment, and the importance for individualized considerations in treatment.

The role of probiotics for ED treatment is also a novel frontier in ED research. In rat models of binge eating and anxiety behaviour, the selective administration of *Bacteroides uniformis* CECT 7771 results in cessation of binge eating and a reduction in anxiety-behaviour [62]. Additionally, randomized control trial comparing the effects of probiotics vs. placebo on 60 adolescent inpatients (ages 13–19) with AN has been planned and the results will glean insight on how probiotics may influence weight gain, ED pathology, and neuropsychological symptoms in adolescents [63]. Like the potential role of FMT to leverage the gut microbiome in ED treatment, probiotic supplementation is an exciting and promising avenue of research.

Additionally, tailoring re-feeding in a manner that leverages the gut microbiome to promote weight gain and decrease ED behaviours may be an effective treatment tool. Specifically, increasing the diversity of microbes may alter dietary preferences and patterns, resulting in weight gain, and repopulating the gut microbiome with organisms that decrease ED-related symptoms—such as *Lactobacilli, Bifidobacterium* spp. and *Enterococcus* spp.,—may result in improved ED recovery rates [64]. As well, restoring the gut microbiota, may correct the dysfunctional physical changes that hinder recovery (e.g. decreased nutrient absorptive capacity), and result in sustained weight gain and improved outcomes [34].

## Limitations and future directions

**Fig. 2:**
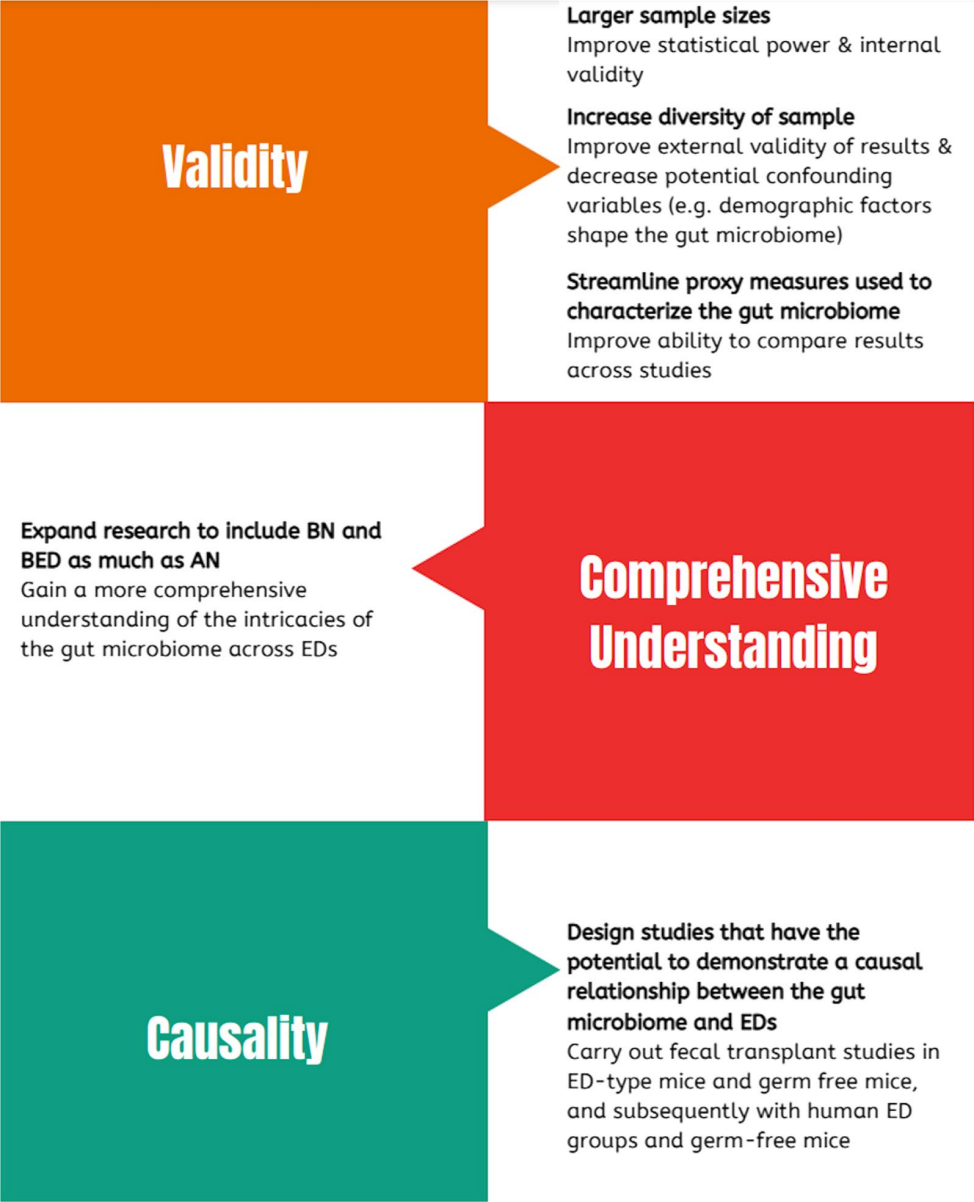
This figure outlines practical steps to move the field of EDs and the gut microbiome forward through: (1) increasing validity of research, (2) developing a more comprehensive understanding of the field, and (3) working towards demonstrating causality

Emerging evidence demonstrates widespread, yet interconnected, relationships between the gut microbiome and various body systems central to EDs. However, current conclusions are speculative and more robust research is needed to prove causation in the relationship between the gut microbiome, the gut-brain axis and EDs. We propose future research focuses on establishing or refuting causality and the subsequent ability to apply the research to clinical practice. We suggest practical steps, outlined in Fig. 2, to work towards this while also addressing the following limitations in the field:

- Current studies rely on small, and relatively homogenous samples, hindering our ability to draw any significant conclusions that can be applied widely.
- Current studies in the field, when done on humans, primarily use ED populations from Western ED treatment centers, resulting in an almost exclusively white, female ED sample.
- Proxy measures used to characterize the gut microbiome differ between studies, limiting our ability to compare outcomes between studies
- The majority of research has been conducted on AN populations, potentially restricting our understanding of the role of the gut microbiome in EDs as other, often co-occurring EDs are not considered. In particular, future research should continue to explore the various subtypes of AN, and should include BN, BED, and ARFID populations.
- Current studies are inconsistent in their designs and the outcome variables cannot suggest causality.

1. Validity: Larger sample sizes are needed to increase the statistical power of the research, and more diversity among the samples would increase the external validity of the research. More diversity is needed within the sample groups as demographic factors are formative in shaping the gut microbiome [65] and without considering these factors the results may be inadvertently confounded. Furthermore, EDs affect individuals of all genders and ethnicities across the globe and a lack of representation in research limits the external validity of the findings. We propose consistent methods are used to characterize the gut microbiome as current studies use different proxy measures to characterize the gut microbiome (e.g. some use α-diversity, others use fecal metabolites), limiting our ability compare findings across studies.
2. Comprehensive understanding: Most research in the ED field has been conducted on AN populations and the research on the gut microbiome in EDs is no exception. To most comprehensively understand the intricacies of the gut microbiome in ED it is important that a spectrum of EDs are considered especially when considering EDs from a transdiagnostic perspective.
3. Causality: Studies have used fecal transplants from humans to germ-free mice demonstrate causality between gut microbes and anthropometric states (e.g. lean vs. obese) [24]. As previous studies have modeled AN in mice, and we propose that the same type of study is first carried out in control mice and AN-mice, and subsequently in human ED groups and germ-free mice, to provide more insight into the relationship between the gut microbiome and EDs, potentially demonstrating causality.

We propose enhanced methodology and more robust studies will propel this field forward. The future of ED treatment could consider FMT to improve recovery rates, continue investigating the role of prebiotics in ED care, and even re-consider refeeding protocols.

## Data Availability

Not applicable.
